# An Analysis of the NSW Midwives Data Collection over an 11-Year Period to Determine the Risks to the Mother and the Neonate of Induced Delivery for Non-Obstetric Indication at Term

**DOI:** 10.1155/2013/178415

**Published:** 2013-09-25

**Authors:** Padmini Raviraj, Aiat Shamsa, Jun Bai, Rajanishwar Gyaneshwar

**Affiliations:** Department of Obstetrics and Gynaecology, Liverpool Hospital, Liverpool, NSW, Australia

## Abstract

*Objective*. To determine the risks of induced term delivery to the mother and neonate at different gestational ages in the absence of obstetric indications. *Study Design*. All deliveries in New South Wales (NSW) between 1998 and 2008 were reviewed from the MDC. Uncomplicated pregnancies which were induced for non-obstetric reasons after 37 completed weeks were reviewed. This was a retrospective, historical cohort study, and both maternal and neonatal outcomes were analysed and compared between different gestational age groups. *Results*. An analysis of the data shows that induction of labour after 37 completed weeks exposes the fetus and mother to different levels of risk at different gestations. *Conclusion*. In an uncomplicated pregnancy, induction of labour is associated with the highest rate of neonatal complication at 37 weeks as compared with rates at later gestations. With each ensuing week, the neonatal outcome improves. At 40 weeks the likelihood of neonatal intensive care admission, low Apgar scores, and perinatal death rate is at its lowest, and then there is a slight but not significant rise after 41 weeks. The likelihood of caesarean section is the lowest when inductions are carried out at 39 weeks and is the highest at 41 weeks and over.

## 1. Introduction

Induction of labour is defined as the artificial initiation of uterine contractions leading to dilatation of the cervix at or after 24 weeks [1]. Induction of labour is relatively safe; hence both clinicians and women feel that this management option normally does not pose serious risk for the mother and the neonate [2]. As a consequence, the rate of this intervention is rising [1, 3]. Induction of labour without a medical indication is termed elective induction. A recent systematic review concludes that elective induction appears to be increasing even more rapidly than inductions, which are indicated on obstetric grounds [4]. 

Inductions based on well-established clinical guidelines are associated with improved maternal and neonatal health outcomes. On the other hand, a number of maternal and neonatal complications, particularly caesarean delivery, have been observed among women induced without a standard clinical indication [2, 5].

A pregnant woman is at “term” when her pregnancy duration reaches 37 completed weeks [6]. New South Wales Health recommends that no elective caesarean deliveries should be carried out before 39 completed weeks because of the risk of neonatal respiratory morbidity [7].

This current study was undertaken to determine complication rates for both mothers and neonates from non-obstetric induction of term pregnancies. These include neonatal Apgar scores, admission to neonatal intensive care unit (NICU), perinatal death and maternal perineal injury, and caesarean section rate. This study has implications for the practice of induction of labour in an otherwise uncomplicated pregnancy.

## 2. Methods

The New South Wales Midwives Data Collection (NSW MDC) is a population based surveillance system covering all births in public and private hospitals as well as home births in NSW Australia [8]. NSW MDC includes demographic details and information of maternal health, pregnancy, labour, delivery, and perinatal outcomes. The accuracy of the data is dependent on the diligence of the attending midwife or doctor completing the standardised notification form when the birth occurs [9]. The period of the data used in this study was between 1998 and 2008. Inductions of labour for gestational age 37 weeks and more where the stated indication was classified as “Other” were analysed. Induction of pregnancy for diabetes, hypertension, proteinuric hypertension, preeclamptic toxaemia, fetal distress, fetal death, chorioamnionitis, isoimmunisation, premature rupture of membranes, and intrauterine growth restriction was excluded from the analysis. A subset of NSW MDC was reviewed to identify those indications classified as “Others.” 

The data was managed and analysed using the SPSS package (SPSS Inc., Chicago, IL, USA). Chi-square tests were used for categorical variables. The *t*-test and analysis of variance were used for continuous variables. Multiple logistic regression analysis was used to adjust for potential confounders. All models were checked for interaction effects. Results are presented as odds ratios (ORs) with the associated 95% confidence interval (95% CI). The independent variables in the initial multiple logistic regression equation were gestational age groups, maternal age, parity, smoking, maternal diabetes, gestational diabetes, and pregnancy induced hypertension. Maternal diabetes was the only other potential confounder for effect of gestational age groups on perinatal mortality.

The study has been approved by South Western Sydney Area Health Service Human Research Ethics Committee.

## 3. Results

During the 11 years of the study period between 1998 and 2008, there were 981,178 births recorded in the NSW MDC. Cases with incomplete information were excluded (*n* = 177). The number of induction of labour was 242,231 (24.7% of all births). Of these inductions, 231,456 were conducted after 37 weeks of gestation (95.5%), among which 151,683 (65.5%) were induced for non-obstetric indications. 

There is a significant (*P* < 0.001) increase in the trend of induction rates through the years, being 24.2% in 1998, reaching 25.2% in 2008. Induction of labour at 37 weeks accounted for 5.4% of all inductions. The rate of induction at 37 weeks appears to have a significantly accelerating trend (*P* < 0.001) over the period studied, rising from 5.2% in 1998 to 6.3% 2008. 

Table 1 presents maternal and delivery characteristics and pregnancy outcomes by gestational age. Neonatal morbidity indicated, by low apgar scores, that NICU admission and perinatal mortality are the best for those inductions carried out at 40 weeks and the worst for those inductions at 37 weeks gestation. Early induction is associated with intact perineum and longer mean length of stay (Table 1). 

Table 2 presents results from the multiple logistic regression models where inductions at 40 weeks of gestation are the reference. Induction at 37 weeks gestation carries 4.6 (95% CI: 2.0–10.2) times more risk of perinatal death. At 38 weeks the risk of perinatal death was 2.4 (95% CI: 1.3–4.4) times greater. The odds ratio of perinatal death at 39 and 41+ weeks gestation, although greater, did not reach statistical significance.

Comparing the risk of admission to NICU, in different gestational age groups, inductions at 37, 38, 39, 41, and more weeks carry higher risk of NICU admission than that at 40 weeks gestation (Table 2).

Induction at 37 weeks and 41+ weeks gestation was associated with increased risk of the Apgar score being less than 5 at 1 minute. The Apgar score at 1 minute was similar for births at 38 weeks and 40 weeks of gestation. Neonates delivered at 39 weeks of gestation had the lowest risk of a low Apgar score at 1 minute (OR: 0.9, 95% CI: 0.8–1.0). Apgar scores of less than 7 at 5 minutes after birth showed a similar trend to that of Apgar scores of less than 5 at 1 minute (Table 2). 

There were lower risks of delivery by caesarean section in women having inductions for non-obstetric reasons at 37, 38, and 39 weeks gestation compared to those women at 40 weeks gestation (Table 2). Inductions after 41 weeks gestation were more likely to require a caesarean section (OR: 1.4, 95% CI: 1.3–1.5).

## 4. Discussion

The purpose of this study is to inform clinicians about the implications of inducing labour for indications other than those, which can be justified on strict clinical grounds.

We are reporting an increasing trend in induction of labour in NSW, which is consistent with the trend in NSW [3]. It is of concern that nearly 25% of all births in NSW were induced. Of these more than a half were for non-obstetrical indications. This indication was covered under the category “Other” in NSW MDC. Whilst we cannot say with certainty that this category has not been contaminated by misclassification, reviewing a subset of this classification, in detail, confirmed that 76% of cases under this category were induced for non-obstetrical indication. We also excluded multiple pregnancies from the analysis. Therefore, whilst, we have been diligent in excluding those factors which might adversely impact on neonatal outcome, we concede that for greater reliability in drawing any conclusions, a prospective study looking at all inductions under the category “Other” is required.

 Inductions for non-obstetric reasons conducted at 37 weeks carry the highest neonatal morbidity and mortality risks. This study shows that inductions for “Other” indications are best carried out at 39 or 40 weeks of gestations, and the neonates delivered after 41+ weeks are also at risk of a compromised outcome. Although the perinatal death rate was low overall, there was a significant decline in neonatal deaths when mothers were induced at 40 weeks gestation. Similar findings were noted with regard to admission to NICU, which was the lowest at 40 weeks. The Apgar scores at 1 and 5 minutes were higher at 40 weeks compared to 37 weeks. This observation confirms the findings from California where acid base status and 5 minute Apgar were compared at different gestations. The results showed that the neonate was least compromised at 40 weeks, and most compromised at 37 weeks, and 41 weeks [10]. According to a prospective cohort study conducted in South Australia, the lowest risk of adverse maternal and infant outcome occurred with birth between 38 and 39 weeks [11]. 

Caesarean section rates in the present study were noted to be higher at 40 weeks and thereafter compared with earlier gestations. This may be due to the fact that induction in women whose cervices remain unfavourable at 40 weeks or more is more likely to fail. More research is needed to study the behaviour of an unfavourable cervix after 40 weeks of gestation.

 Heffner et al. [12] had similar findings in their study and concluded that gestational age beyond 40 weeks at induction of labour independently increases the risk of caesarean delivery for both nulliparous and parous women.

The present study confirms the observations of a well-designed prospective study by Clark et al. [13]. Grobman [14] has also concluded that there is an increased risk of adverse neonatal outcome and caesarean sections rate in elective deliveries before completion of 39 weeks. These studies confirm the wisdom of the recommendations of professional bodies such as the American College of Obstetricians and Gynaecologists and their British and Canadian counterparts. They recommend that elective term deliveries should be restricted to a confirmed gestational age of at least 39 weeks unless there are psychological or social reasons and the woman has a favourable cervix. Inspite of the evidence supporting these recommendations they seem however to be disregarded on many occasions [15]. 

These present and other recent observations on deliveries at 37 completed weeks and the associated neonatal risks should logically question the wisdom of defining term as 37 completed weeks of gestation as at this gestation the fetus remains vulnerable. Such a definition may lead obstetricians to have a false sense of security in recommending an early delivery for social or other reasons consequently jeopardising the neonate. 

This study has several limitations. The study does not take the Bishop's score at induction into account. It also has relied on the accuracy of the MDC for classification of the indications for induction. We are aware that information gathered by excluding categories runs the risk of bias due to misclassification. This may have occurred in the MDC notifications. 

Within these limitations, this study informs the clinician about the potential neonatal risks of inducing labour at gestational ages after 37 weeks. 

## Figures and Tables

**Table 1:** Outcomes of induction with non-obstetric indication by different gestational age groups.

Outcomes	Gestational Age group						*P*-value^†^
	37 weeks	38 weeks	39 weeks	40 weeks	≥41 weeks	Total	
Maternal mean age (years)	30.1	30.9	31.1	30.7	29.5	30.2	<0.001
Primigravida	984/3192 (30.8%)	3502/13930 (25.1%)	6046/21391 (28.3%)	16054/39973 (40.2%)	39705/73133 (54.3%)	66291/151619 (43.7%)	<0.001
Maternal Smoking	529/3179 (16.6%)	1885/13915 (13.5%)	2530/21369 (11.8%)	4761/39931 (11.9%)	10690/73021 (14.6%)	20395/151415 (13.5%)	<0.001
Normal Vaginal	2524/3194 (79.0%)	10820/13934 (77.7%)	16191/21391 (8.2%)	27766/39965 (69.5%)	46741/73132 (63.9%)	104042/151616 (68.6%)	<0.001
Forceps	94/3194 (2.9%)	470/13934 (3.4%)	1043/21391 (4.9%)	2523/39965 (6.3%)	4189/73132 (5.7%)	8319/151616 (5.5%)	<0.001
Vacuum Extraction	190/3194 (5.9%)	1145/13934 (8.2%)	1736/21391 (8.1%)	3804/39965 (9.5%)	7114/73132 (9.7%)	13989/151616 (9.2%)	<0.001
Caesarean Section	377/3195 (11.8%)	1454/13942 (10.4%)	2362/21398 (11%)	5799/39984 (14.5%)	15009/73164 (20.5%)	25001/151683 (16.5%)	<0.001
Intact Perineum	1002/2301 (43.5%)	3997/10030 (39.9%)	5730/15510 (36.9%)	11066/31163 (35.5%)	21176/56479 (37.5%)	42971/115483 (37.2%)	<0.001
Admitted to NICU	81/2301 (3.5%)	153/10030 (1.5%)	175/15509 (1.1%)	236/31161 (0.8%)	613/56477 (1.1%)	1258/115478 (1.1%)	<0.001
Postpartum Haemorrhage	11/893 (1.2%)	60/3910 (1.5%)	115/5883 (2.0%)	167/8814 (1.9%)	347/16675 (2.1%)	700/36175 (1.9%)	>0.05
Surgical Repair of Vagina or Perineum	1373/3194 (43.0%)	6964/13937 (50.0%)	11594/21394 (54.2%)	22309/39978 (55.8%)	37792/73150 (51.7%)	80032/151653 (52.8%)	<0.001
Apgar < 5 at 1 Minute	127/3191 (4.0%)	396/13935 (2.8%)	572/21392 (2.7%)	1227/39971 (3.1%)	3228/73143 (4.4%)	5550/151632 (3.7%)	<0.001
Apgar < 7 at 5 Minutes	79/3191 (2.5%)	180/3191 (1.3%)	230/21391 (1.1%)	474/39967 (1.2%)	1176/73144 (1.6%)	2139/151630 (1.4%)	<0.001
Still birth	5/2300 (0.22%)	10/10028 (0.09%)	9/15506 (0.06%)	14/31162 (0.04%)	30/56470 (0.05%)	68//115347 (0.06%)	<0.001
Neonatal Death	3/2296 (0.13%)	8/10020 (0.08%)	3/15501 (0.02%)	9/31149 (0.03%)	28/56449 (0.05%)	51/115415 (0.04%)	<0.001
Perinatal Death	8/2300 (0.35%)	18/10028 (0.18%)	12/15506 (0.08%)	23/31162 (0.07%)	58/56470 (0.10%)	119/115347 (0.10%)	<0.001
Birth Weight (grams)	3153	3386	3526	3646	3724	3632	<0.001
Birth Weight < 2,500 grams	174/3192 (5.5%)	215/13937 (1.5%)	121/73144 (0.6%)	99/39973 (0.2%)	121/73144 (0.2%)	730/151630 (0.5%)	<0.001
Mean Length of Stay of Baby (days)	3.9	3.7	3.6	3.7	3.5	3.6	<0.001

^†^ *P*-value for overall difference.

**Table 2:** Risks of induced term delivery to the mother and baby by gestational age.

Outcomes	*n*/*N* (%)	Adjusted OR (95% CI)
Perinatal Death^†^		
37 weeks Gestation	8/2300 (0.3%)	4.57 (2.04–10.24)
38 weeks Gestation	18/10028 (0.2%)	2.39 (1.29–4.44)
39 weeks Gestation	12/15506 (0.1%)	1.04 (0.52–2.08)
40 weeks Gestation (reference)	23/31162 (0.1%)	1
41 weeks Gestation and after	58/56470 (0.1%)	1.39 (0.86–2.26)
Admission to NICU^‡^		
37 weeks Gestation	81/2301 (3.5%)	4.75 (3.67–6.16)
38 weeks Gestation	153/10030 (1.5%)	2.11 (1.71–2.59)
39 weeks Gestation	175/15509 (1.1%)	1.54 (1.27–1.88)
40 weeks Gestation (reference)	236/31161 (0.8%)	1
41 weeks Gestation and after	613/56477 (1.1%)	1.36 (1.17–1.59)
Apgar min 1 < 5^§^		
37 weeks Gestation	127/3191 (4%)	1.35 (1.12–1.62)
38 weeks Gestation	396/13935 (2.8%)	0.96 (0.86–1.08)
39 weeks Gestation	572/21392 (2.7%)	0.9 (0.81–0.99)
40 weeks Gestation (reference)	1227/39971 (3.1%)	1
41 weeks Gestation and after	3228/73143 (4.4%)	1.41 (1.31–1.5)
Apgar min 5 < 7^*∂*^		
37 weeks Gestation	79/3191 (2.5%)	2.22 (1.74–2.82)
38 weeks Gestation	180/3191 (1.3%)	1.17 (0.98–1.39)
39 weeks Gestation	230/21391 (1.1%)	0.95 (0.81–1.11)
40 weeks Gestation (reference)	474/39967 (1.2%)	1
41 weeks Gestation and after	1176/73144 (1.6%)	1.3 (1.16–1.44)
Likelihood of Caesarean Section^¶^		
37 weeks Gestation	377/3195 (11.8%)	0.94 (0.82–1.08)
38 weeks Gestation	1454/13942 (10.4%)	0.89 (0.82–0.96)
39 weeks Gestation	2362/21398 (11%)	0.87 (0.82–0.93)
40 weeks Gestation (reference)	5799/39984 (14.5%)	1
41 weeks Gestation and after	15009/73164 (20.5%)	1.39 (1.34–1.45)

^†^Adjusted for potential confounder of gestational diabetes. All inductions for diabetes, hypertension, fetal distress, fetal death, chorioamionitis, isoimmunisation, PROM, and IUGR were excluded. ^‡^Adjusted for parity, smoking, maternal diabetes, gestational diabetes, and pregnancy induced hypertension. Subjects were singletons without indications of induction diabetes, hypertension, fetal distress, fetal death, chorioamionitis, isoimmunisation, PROM, and IUGR. ^§^Adjusted for maternal age, smoking, parity, diabetes, and gestational diabetes. Subjects with indications of induction excluded were diabetes, hypertension, fetal distress, fetal death, chorioamionitis, isoimmunisation, PROM, and IUGR. ^∂^Adjusted for parity, diabetes, and gestational diabetes. Subjects were singletons without indications of induction of diabetes, hypertension, fetal distress, fetal death, chorioamionitis, isoimmunisation, PROM, and IUGR. ^¶^Adjusted for maternal age, parity, smoking, maternal hypertension, pregnancy induced hypertension, maternal diabetes, and gestational diabetes. Subjects were singletons without indications of induction of diabetes, hypertension, fetal distress, fetal death, chorioamionitis, isoimmunisation, PROM, and IUGR.
